# Low genetic diversity and complexity of submicroscopic *Plasmodium falciparum* infections among febrile patients in low transmission areas in Senegal

**DOI:** 10.1371/journal.pone.0215755

**Published:** 2019-04-25

**Authors:** Rokhaya Sane, Cheikh Talla, Babacar Diouf, Fatoumata Diene Sarr, Nafissatou Diagne, Joseph Faye, Abdoulaye Badiane, Pape Mbacké Sembène, Cheikh Sokhna, Aissatou Toure-Balde, Makhtar Niang

**Affiliations:** 1 Institut Pasteur Dakar, Immunology Unit, Dakar, Sénégal; 2 Département de Biologie Animale, Faculté des Sciences et Techniques, Université Cheikh Anta Diop de Dakar, Dakar Fann, Sénégal; 3 Institut Pasteur Dakar, Epidemiology of Infectious Diseases Unit, Dakar, Sénégal; 4 VITROME, Campus International IRD-UCAD, Dakar, Sénégal; Instituto Rene Rachou, BRAZIL

## Abstract

**Introduction:**

Submicroscopic *Plasmodium* infections are common in malaria endemic countries, but very little studies have been done in Senegal. This study investigates the genetic diversity and complexity of submicroscopic *P*. *falciparum* infections among febrile patients in low transmission areas in Senegal.

**Materials and methods:**

Hundred and fifty blood samples were collected from febrile individuals living in Dielmo and Ndiop (Senegal) between August 2014 and January 2015, tested for microscopic and sub-microscopic *P*. *falciparum* infections and characterized for their genetic diversity and complexity of infections using *msp-1* and *msp-2* genotyping.

**Results:**

Submicroscopic *P*. *falciparum* infections were 19.6% and 25% in Dielmo and Ndiop, respectively. K1 and 3D7 were the predominant *msp-1* and *msp-2* allelic types with respective frequencies of 67.36% and 67.10% in microscopic isolates and 58.24% and 78% in submicroscopic ones. Frequencies of *msp-1* allelic types were statistically comparable between the studied groups (p>0.05), and were respectively 93.54% vs 87.5% for K1, 60% vs 54.83% for MAD20 and 41.93% vs 22.5% for RO33 while frequencies of *msp-2* allelic types were significantly highest in the microscopy group for FC27 (41.93% vs 10%, Fisher’s Exact Test, p = 0.001) and 3D7 (61.29% vs 32.5%, Fisher’s Exact Test, p = 0.02). Multiplicities of infection were lowest in submicroscopic *P*. *falciparum* isolates.

**Conclusions:**

The study revealed a high submicroscopic *P*. *falciparum* carriage among patients in the study areas, and that submicroscopic *P*. *falciparum* isolates had a lower genetic diversity and complexity of malaria infections.

## Background

The scale-up of malaria control interventions has substantially reduced malaria burden and transmission across several malaria endemic countries over the past 15 years with the number of malaria infections and malaria-related mortality that dropping significantly [1]. In Senegal, similar results were obtained with a globally significant reduction of the proportional malaria-related morbidity and mortality from 35.72% and 28.72% in 2001 to 28.72% and 1.73% in 2017, respectively despite significant disparities between regions [2]. In fact, while malaria elimination strategies have been implemented in the North, applied preventive and control measures had limited impact in the South and Southeast regions where malaria transmission is still active [2].

All malaria control programs have passive surveillance systems thatidentify and treat individuals with light microscopy (LM) and/or rapid diagnostic test (RDT)-confirmed malaria diagnosis at health facilities. In most settings, quality assured LM and RDT effectively diagnose the majority of symptomatic patients, thus guiding treatment. However, with the continuous decline of malaria transmission and progress towards elimination, both LM and RDT lack sufficient sensitivity to detect low-density parasitaemia, leading to an underestimation of parasite prevalence and malaria-related morbidity where infections are mostly subpatent in many areas [3,4,5]. The rapid advances in nucleic acid testing regularly used in research settings have led to highly sensitive, specific and quantitative molecular diagnosis for malaria, and have increasingly revealed the widespread presence of low-density submicroscopic *Plasmodium* infections in both febrile and asymptomatic subjects [3,5,6,7]. Studies have estimated that submicroscopic *Plasmodium* infections sourced for 20–50% of all human-to-mosquito transmissions when transmission reaches very low levels [8], and a meta-analysis of community-based study has shown that microscopy only detected 50% of the infections identified by PCR [9]. Together, this revealed the importance of submicroscopic *Plasmodium* infections for ongoing malaria transmission and highlighted the relevance of addressing such infections.

Submicroscopic *Plasmodium* carriage has been reported from different regions of the globe in febrile and asymptomatic individuals including Senegal [7,10,11]. While several human-related factors known to protect against clinical malaria, have been associated with low-density parasite carriage [12,13,14]; parasites may also have varying propensities to remain at low density when infecting hosts, for example through reduced multiplication rates [15]. The intensity of malaria transmission has been shown to mirror the level of *P*. *falciparum* genetic diversity and population structure with a low transmission intensity correlated with a lower genetic diversity of *Plasmodium* isolates [16,17,18].

To date, very little data is available on the genetic diversity of submicroscopic *P*. *falciparum* isolates in Senegal. In this study, the prevalence of submicroscopic *P*. *falciparum* infections was explored among febrile patients during the malaria transmission season in low transmission areas in Senegal. In addition, genotyping of *msp-1* and *msp-*2 polymorphic markers was used to examine the genetic diversity and complexity of submicroscopic *P*. *falciparum* infections.

## Methods

### Study sites, design and samples collection

Samples used in this study were collected from patients living in Dielmo and Ndiop Senegalese villages, which have been extensively described. Written informed consent was obtained from adults and legal guardians of minors participants as part of the ongoing malaria surveillance programs in the two villages[19,20,21,22,23]. The Ndiop and Dielmo project was initially approved by the Senegalese National Health Research Ethics Committee and was renewed on a regular basis.

For this study, 150 patients from Dielmo (N = 102) and Ndiop (N = 48) presenting with febrile syndromes and attending Dielmo and Ndiop health facilities between August 2014 and January 2015 were enrolled. Venous blood (5 mL) were collected for determination of microscopic and submicroscopic *Plasmodium* parasites carriage. Genomic DNA (gDNA) from mono-infected *P*. *falciparum* positive samples were genotyped using *msp-1* (block 2) and *msp-2* (block 3) polymorphic markers as described previously [24] to investigate and compare the genetic diversity and complexity of *P*. *falciparum* infections between microscopic and sub-microscopic parasites populations.

### Determination of microscopic and submicroscopic malaria parasites carriage

Microscopic *Plasmodium* infections were determined by light microscopy examination of thin and thick smears systematically prepared on site at the health facilities. Blood smears were prepared, air-dried, fixed in methanol and stained with Giemsa for 20–30 minutes. At least two hundred oil-immersion fields were examined at 100X magnification before a slide was considered negative. A second confirmatory reading was performed later at the Institute for Research and Development, and the results herein presented refer only to concordant data between the two readings.

The presence of *Plasmodium* spp was further investigated at the laboratory by qPCR to distinguish microscopic from submicroscopic *Plasmodium* infections. To this end, genomic DNA (gDNA) isolated using QIAamp DNA Blood Mini Kit (Qiagen, Hilden, Germany) according to manufacturer’s instructions was subjected to a two-step real time PCR with genus- and species-specific primers targeting the *Plasmodium* Cytochrome B gene as previously described [25,26]. DNA of confirmed *P*. *falciparum*, *P*. *malariae*, *P*. *ovale* and *P*. *vivax* infected patients were used as positive controls in all assays, and water and DNA from malaria negative individual served as negative controls to ensure lack of contamination [27].

Submicroscopic *Plasmodium* carriage was defined as an infection with any *Plasmodium* detected by qPCR but not by microscopy.

### Genotyping of *Plasmodium falciparum* isolates

Genotyping of both microscopic and submicroscopic *P*. *falciparum* isolates was carried out by nested PCR amplification of the two highly polymorphic regions of *msp-1* (block 2) and *msp-2* (block 3) genes as reported previously [24,28]. Primer sequences and cycling parameters used for amplification of the three allelic families of *msp-1* (K1, MAD20 and RO33) and two allelic families of *msp-2* (FC27 and 3D7) have been reported elsewhere [24]. Amplified products were separated by electrophoresis on 1.5% ethidium bromide-stained agarose gel, and the fragments were visualized under UV light. The sizes of amplified products were determined using a Hyperladder 1kb (Bioline) molecular weight marker.

The number of *P*. *falciparum* isolates with at least one *msp* allele out of the total number of isolates was used to calculate the frequency of each *msp* allelic family. The total number of fragments detected in *msp-1* or *msp-2* was divided by the number of isolates positive for the same marker to define the number of genotypes per infection or mean multiplicity of infections (MOI) as described earlier [29,30].

### Statistical analysis

R statistical software version 3.3.1 was used for data analysis [31]. Distributions of age and sex between microscopic and submicroscopic groups were compared using Kruskall Wallis rank sum tests. Fisher’s Exact tests and Exact Binomial test were used to compare the respective frequencies of unique allelic families and combination of allelic families between the two study groups. The relationship between age and allelic frequencies between the two groups was estimated using Kruskal Wallis non-parametric test. The p-value of less than or equal to 0.05 indicates a statistically significant difference.

## Results

### Baseline characteristics of the study population

Demographic details of the 150 febrile patients enrolled for the study are given in Table 1. Proportions of male (51.33%) and female patients (48.67%) were comparable, and the mean age was 15.05 years [range 0.4–92.8 years] and 13.81 years [range 1.5–59.1 years] in Dielmo and Ndiop, respectively. The majority of patients were children under 5 years old in Dielmo (39.22%), and older children aged 5–15 years old (43.74%) in Ndiop (Table 1).

**Table 1:** Baseline characteristics of the enrolled population.

	Dielmo		Ndiop		Total
Total	N = 102		N = 48		N = 150
	N	%	N	%	
**Sex**					
Male	60	58.82 [49–68]	17	35.42 [22–51]	77
Female	42	41.18 [32–51]	31	64.58 [49–78]	73
**Age**					
Mean	15.05	**–**	13.81	**–**	14.43
Range	[0.4–92.8]	**–**	[1.5–59.1]	**–**	[0.4–92.8]
**Age groups**					
≤ 5 years	40	39.22 [30–49]	13	27.08 [15–42]	53
]5–15] years	28	27.45 [19–37]	21	43.75 [29–59]	49
>15 years	34	33.33 [24–43]	14	29.16 [17–44]	48

### Microscopic and submicroscopic *P*. *falciparum* carriage

Of the 150 blood samples screened for the presence of malaria parasites, 20.66% (31/150) were positive for *P*. *falciparum* by microscopy while qPCR-based molecular screening detected *P*. *falciparum* DNA in 47.33% (71/150) of samples. The latter comprised all 31 microscopy positive samples and 40 microscopy negative samples (submicroscopic *P*. *falciparum* carriage). The specific prevalence of submicroscopic *P*. *falciparum* carriage among the enrolled population was 26.66% (40/150).

The demographic characteristics of microscopic and submicroscopic *P*. *falciparum*-infected patients subsequently analyzed in this study for their genetic diversity and complexity of *P*. *falciparum* infections are detailed in Table 2. The median age of patients was 14.7 years and 6.85 years for the microscopy and submicroscopy groups, respectively (Table 2). Patients aged ≤15 years old represented 51.6% and 70% in microscopy and submicroscopy groups, respectively (Table 2). The sex ratios (M/F) were in favor of males (M/F = 1.38) and females (M/F = 0.73) in the microscopy and submicroscopy groups respectively (Table 2). Patients from both groups were comparable in term of sex (Fisher’s Exact test, p = 0.24) but differed in term of age (Kruskall Wallis rank sum test, p = 0.02) (Table 2).

**Table 2:** Demographic characteristics of the study population.

	Microscopy		Sub-microscopy		P-value
Total	N = 31		N = 40		
	N	(%)	N	(%)	
**Sex**					
Male	18	58.06	17	42.5	
Female	13	41.94	23	57.5	0.24
Ratio (M/F)	1.38	-	0.73	-	
**Age (years)**					
Median	14.7	**–**	6.85	**–**	0.02
IQ*Range	17.1	**–**	19.6	**–**	
**Age groups**					
≤ 15 years	16	51.6	28	70	
>15 years	15	48.4	12	30	
**Location**					
Dielmo	17	54.83	28	70	
Ndiop	14	45.16	12	30	

*IQ: interquartile

### Genetic diversity of microscopic and submicroscopic *P*. *falciparum* isolates

**Fig 1:**
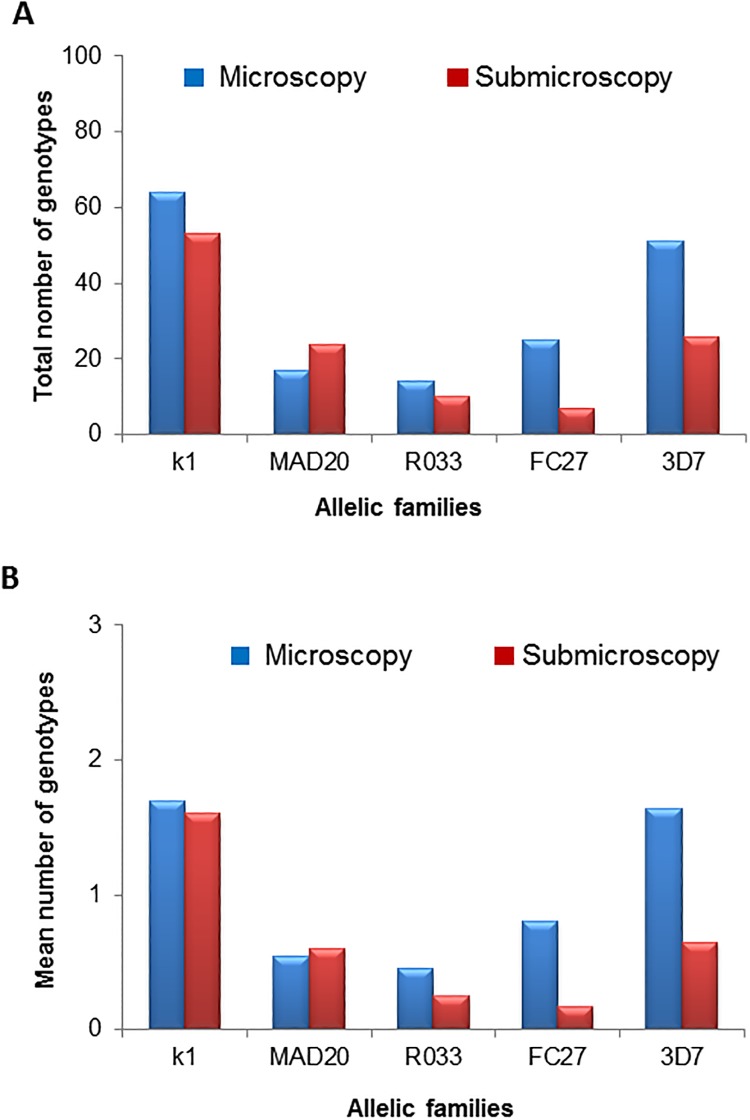
Comparison between total (A) and mean (B) number of genotypes per allelic family between microscopy and submicroscopy groups.

Microscopic and submicroscopic *P*. *falciparum* isolates were successfully genotyped for both *msp-1* and *msp-2* polymorphic markers and their respective three (K1, MAD20 and RO33) and two (3D7 and FC27) allelic families were detected at various proportions in both groups. The proportions of positive *msp-1* and *msp-2* samples were 80.66% (25/31) and 90.32% (28/31) for the microscopy group and 87.5% (35/40), and 70% (28/40) for the submicroscopy group. The genetic diversity of microscopic and submicroscopic *P*. *falciparum* isolates was compared using mean numbers of *msp-1* and *msp-2* genotypes instead of the total number of genotypes to rule out the potential impact of the highest number of submicroscopic *P*. *falciparum* isolates on the genetic diversity. This revealed comparable mean numbers of *msp-1* genotypes between microscopic and submicroscopic *P*. *falciparum* isolates (1.7 vs 1.6 for K1, 0.54 vs 0.6 for MAD20 and 0.45 vs 0.25 for RO33) (Fig 1). In contrast to *msp-1* locus, the mean numbers of *msp-2* genotypes were significantly higher in microscopy-detectable *P*. *falciparum* isolates and were 0.8 vs 0.17 for FC27 (Fisher’s Exact test, p<0.05) and 1.64 vs 0.65 for 3D7 (Fisher’s Exact test, p = 0.02) (Fig 1). These observations support a low genetic diversity of submicroscopic *P*. *falciparum* isolates from febrile patients analyzed in this study.

#### Frequency of *msp-1* and *msp-2* allelic families

**Fig 2:**
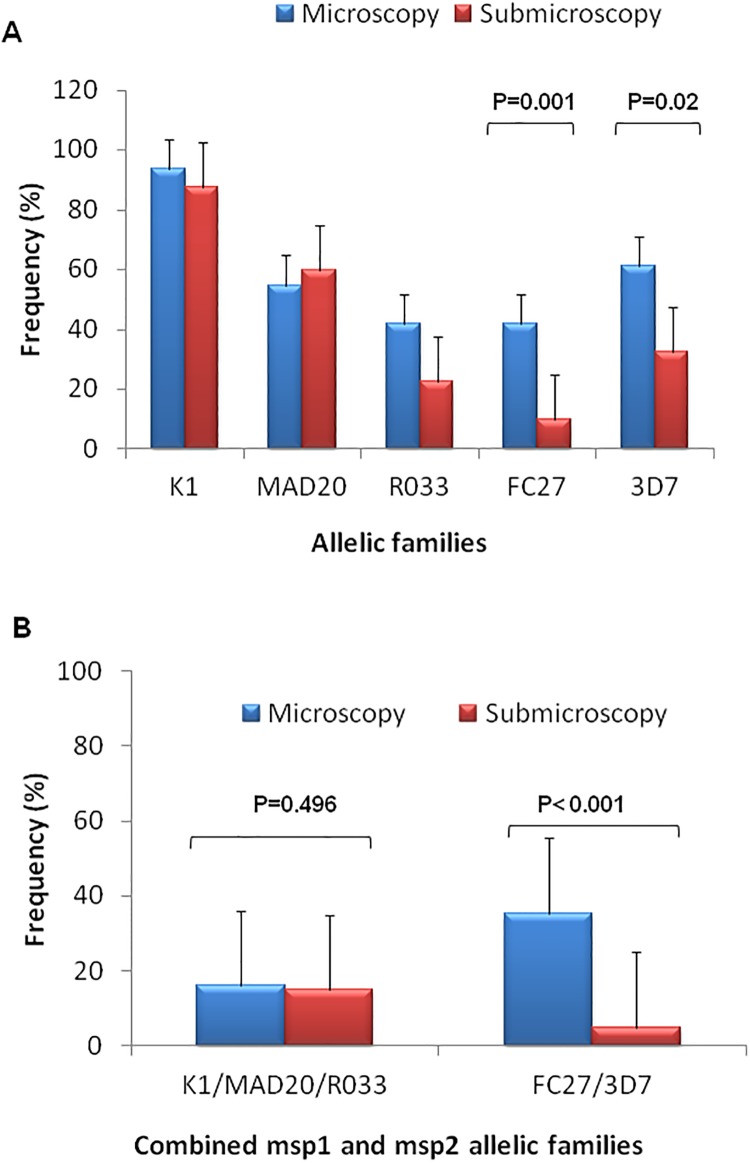
Frequency of unique (A) and combined (B) *msp-1* and *msp-2* allelic families.

The frequency of K1, MAD20 and RO33 allelic families of *msp-1* and 3D7 and FC27 allelic families of *msp-2* were compared between microscopic and submicroscopic *P*. *falciparum* isolates. K1 and 3D7 allelic families were the respective predominant *msp-1* and *msp-2* allelic types in both microscopic and submicroscopic *P*. *falciparum* isolates (Fig 2A). Frequencies of *msp-1* specific allelic types were statistically comparable (Fisher’s exact test, p>0.05) between microscopic and submicroscopic *P*. *falciparum* isolates and were respectively 93.54% vs 87.5% for K1, 60% vs 54.83% for MAD20 and 41.93% vs 22.5% for RO33 (Fig 2A). Carriage of trimorphic *msp-1* allelic combinations (K1/MAD20/RO33) accounted for 16.12% and 15% of *msp-1* positive isolates from microscopic and submicroscopic isolates respectively with no statistically significant difference (Binomial exact test, p = 0.49) (Fig 2B).

By contrast to *msp-1* allelic types, the frequencies of *msp-2* specific allelic types were significantly higher in microscopy-detectable *P*. *falciparum* isolates than submicroscopic ones and were respectively 41.93% vs 10% for FC27 (Fisher’s exact test, P = 0.001) and 61.29% vs 32.5% for 3D7 (Fisher’s exact test, P = 0.02) (Fig 2A). Dimorphic FC27/3D7 allelic combination was significantly different (Binomial exact test, p = 0.001) between microscopic (35.48%) and submicroscopic (5%) *P*. *falciparum* isolates (Fig 2B).

**Fig 3:**
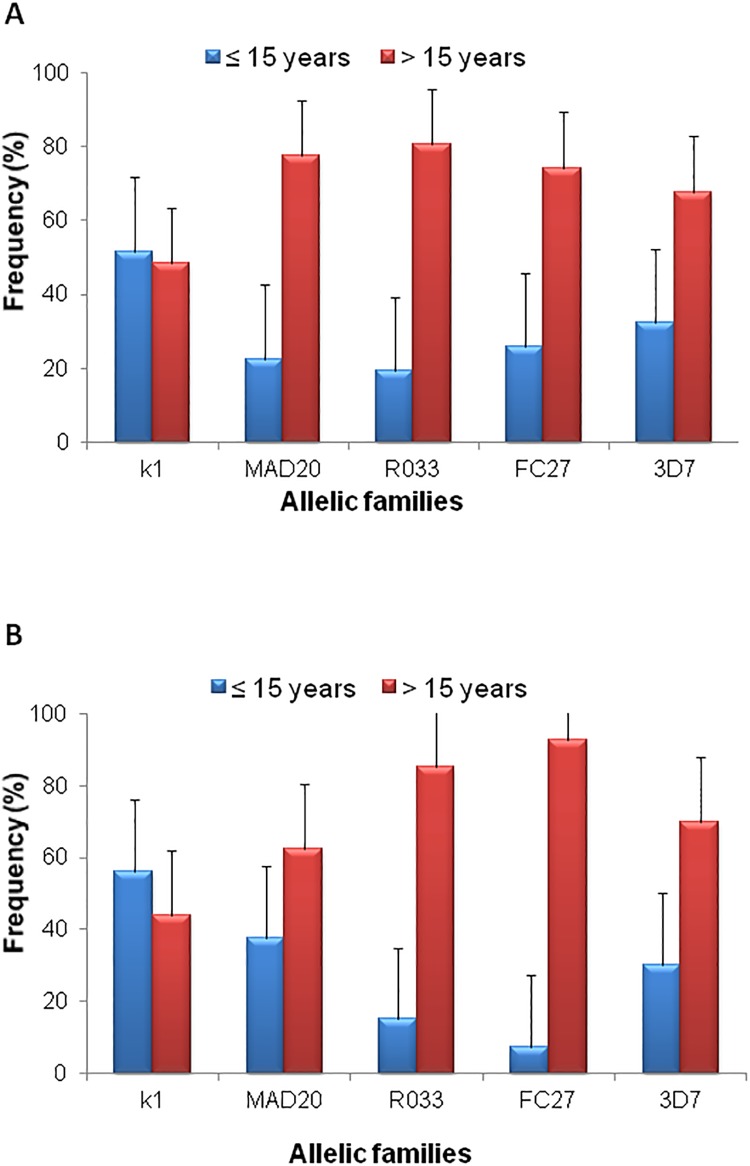
Distribution of *msp-1* and *msp-2* alleles by age in microscopy (A) and submicroscopy (B) groups.

Frequencies of specific *msp-1* and *msp-2* allelic families were also analyzed with respect to two arbitrary defined age groups: ≤ 15 years and > 15 years (Table 2). The analysis revealed no preferential carriage of K1 allelic type between the two age groups for both microscopic (Fig 3A) and sub-microscopic (Fig 3B) *P*. *falciparum* isolates. In contrast, MAD20 and RO33 *msp-1*-allelic types and the two allelic families of *msp-2* (FC27 and 3D7) were significantly more abundant (Fisher’s exact test, p<0.05) in *P*. *falciparum* isolates from patients >15 years in both studied groups (Fig 3A and 3B).

#### Mean multiplicity of *Plasmodium falciparum* infections

Numerous samples showed several distinct amplified fragments, indicating the presence of more than one genotype per infection. The mean multiplicity of *P*. *falciparum* infections (MOI) was found to be 2.12 and 1.60 respectively for *msp-1* and *msp-2* in the microscopy group (Table 3). In the sub-microscopy group, the mean MOI was 1.7 and 1.03 for *msp-1* and *msp-2*, respectively (Table 3).

**Table 3:** Distribution of the MOI by age groups and villages.

		Mean MOI			
		Microscopy		Sub-microscopy	
		*msp-1*	*msp-2*	*msp-1*	*msp-2*
**General**		2.12	1.60	1.7	1.32
**Age group**					
	**≤ 15 years**	1.88	1.74	1.29	1.45
	**> 15 years**	2.00	1.46	1.9	1.20
**Villages**					
	**Dielmo**	2.23	1.58	1.16	1.54
	**Ndiop**	2.00	1.92	1.82	1.10

The distribution of both *msp-1* and *msp-2* mean MOI according to the two age groups showed no association with age for both studied groups (Table 3).

The analysis of mean MOI in relation to village of origin of patients (Dielmo vs Ndiop) showed no significant difference neither with *msp-1*, nor with *msp-2* (Table 3).

## Discussion

To date, very few studies have investigated the genetic structure of submicroscopic malaria parasites populations circulating in endemic countries. Our study aimed at filling this major gap and evaluated the genetic diversity, allelic frequency of *msp-1* and *msp-2*, and complexity of submicroscopic *P*. *falciparum* infections in isolates from patients in Dielmo and Ndiop, two low-transmission areas in Senegal.

The study revealed a high proportion of submicroscopic *P*. *falciparum* carriage among febrile patients in both villages, thus highlighting the limitations of LM to accurately diagnose *Plasmodium* infections when approaching elimination. Controlled malaria infection studies have shown that most patients experienced fluctuations of parasite densities under and over the microscopic detection threshold, thus leading to alternative periods of missed and detectable submicroscopic infections [32]. The reduced parasite densities below the microscopic detection threshold is likely to reflect an anti-parasitic acquired immunity but can also be linked to human genetic traits that favor low-density malaria parasites carriage. Whatever the case, further investigations are needed to confirm such hypotheses.

The importance of low-density infection as a public health concern is supported by studies that have shown that mosquitoes feeding on individuals with parasite-negative blood smears can become infected with malaria [33]. Moreover, both theoretical and experimental data supported a considerable probability of malaria transmission occurring at parasite densities missed by LM and RDT [34]. Thus, submicroscopic *Plasmodium* infections constitute a challenge to control program aiming to achieve elimination, and should be tackled with the deployment of more sensitive point-of-care diagnostics.

The study also revealed a lower genetic diversity of submicroscopic *P*. *falciparum* isolates compared to microscopic isolates. An explanation to this could be due to the parasite densities in the submicroscopic infections, which are low and less likely to amplify. However, the high proportions of positive *msp-1* and *msp-2* samples observed in both microscopy and submicroscopy groups strongly suggest that parasite densities were not the only cause of the lower genetic diversity observed in submicroscopic *P*. *falciparum* isolates. The predominance of K1 and 3D7 for *msp-1* and *msp-2* observed in this study was also demonstrated in other studies from West Africa including Burkina Faso [35,36], Cote d’Ivoire [37]and Gabon [37], as well as Senegal [38,39]. The finding that there are no differences in frequencies in the *msp-1* allelic families but lower frequencies in the *msp-2* allelic families among the sub-microscopic group is interesting. This could be explained by the highest discriminatory power of *msp-2* polymorphic marker over *msp-1* marker, thus justifying the widest use of *msp-2* marker many genetic diversity studies and to distinguish recrudescence versus re-infection in drug resistance studies[30,40,41,42].

For both *msp-1* and *msp-2* loci, the means MOI was lowest in submicroscopic *P*. *falciparum* isolates, thus supporting a direct relationship between mean MOI and parasite density as reported in several others studies [43,44,45]. Previous studies have shown a pattern of greater MOI in older individuals than younger individuals probably reflecting more previous exposure to infection [46,47]. However, conflicting findings showing decreased MOI with age were also reported [30,48]. Studies that focused on *msp-2* typing based on its highest discriminatory power have shown a significant decrease of mean MOI with increasing age and the age-effect was most marked in subjects older than 17 years [41,49]. In this present study, *msp-1-*based mean MOI increased with age in both studied groups contrasting with *msp-2*-based mean MOI, which decreased with age of patients in both studied groups. The latter finding agrees with a study in Senegal where the number of genotypes decreased with age in those aged 15 and above [50]. The findings are also in agreement with studies that have shown positive correlations between parasite density and MOI in infants and young children but not in older individuals [50,51] since the majority of our study populations comprised infants and young children aged 15 years and below in both microscopy (51.6%) and sub-microscopy (70%) groups. The contrasting findings revealed by the different studies depicted the complex nature of the relationship between mean MOI and age.

The low MOI observed in this study, in particular for the submicroscopic *P*. *falciparum* parasites is explained by the fact that only one fragment was generated from most of the positive samples while some of the microscopic *P*. *falciparum* parasites produced two or three amplicons.

Agarose gel electrophoresis of *msp-1* and *msp-2* amplified products lacks sufficient sensitivity to resolve very small size difference between different bands, which thus constitute a limitation of the study. Alternative methods such as genotyping using capillary electrophoresis or whole genome sequencing are preferred and offer better resolution to distinguish parasite populations.

Taken together, the results reported in this study indicate a low genetic diversity and MOI of submicroscopic *P*. *falciparum* isolates in febrile patients. This establishes a correlation between the genetic diversity, MOI and parasitaemia as described by others [52,53,54] and provides an explanation of the lower genetic diversity and MOI of microscopically undetectable *P*. *falciparum* isolates.

## Conclusions

The findings revealed a high proportion of submicroscopic *P*. *falciparum* carriage among patients and a low genetic diversity and MOI associated with submicroscopic *P*. *falciparum* infections.

A deeper understanding of the genetic characteristics of low-density submicroscopic *Plasmodium* parasites along with the investigation of human genetic factors that contribute to the maintenance of low-density *P*. *falciparum* parasites are critically needed to guide targeted interventions on the road to malaria elimination.
